# Distinct associations between body mass index and polyunsaturated fatty acids in dementia with Lewy Bodies and Alzheimer’s disease

**DOI:** 10.1016/j.jnha.2026.100810

**Published:** 2026-02-21

**Authors:** Nicolás Castellanos-Perilla, Miguel Germán Borda, Joyce Ruifen Chong, Diego Alejandro Tovar-Rios, Alberto Jaramillo-Jimenez, Rolf K Berge, George E. Barreto, Dag Aarsland, Audun Osland Vik-M

**Affiliations:** a Department of Clinical Medicine, University of Bergen, Bergen, Norway; b Centre for Age-Related Medicine (SESAM), Stavanger University Hospital, Stavanger, Norway; c Department of Neurobiology, Care Sciences and Society, Karolinska Institutet, Stockholm, Sweden; d Department of Neurology, Clínica Universidad de Navarra, Pamplona, Spain; e Department of Pharmacology, Yong Loo Lin School of Medicine, National University of Singapore, Singapore, Singapore; f Memory, Aging and Cognition Centre, National University Health Systems, Singapore, Singapore; g Centre for Healthy Brain Ageing, Institute of Psychiatry, Psychology, and Neuroscience, King's College London, London, United Kingdom; h L-BioStat, KU Leuven, Leuven, Belgium; i Grupo de Investigación en Estadística Aplicada - INFERIR, Facultad de Ingeniería, Universidad del Valle, Santiago de Cali, Valle del Cauca, Colombia; j Prevención y Control de la Enfermedad Crónica - PRECEC, Facultad de Salud, Universidad del Valle, Santiago de Cali, Valle del Cauca, Colombia; k Grupo de Neurociencias de Antioquia (GNA), Facultad de Medicina, Universidad de Antioquia, Medellín, Colombia; l Department of Clinical Science, University of Bergen, Bergen, Norway; m Department of Biological Sciences, University of Limerick, Limerick, Ireland

**Keywords:** Dementia, neurodegenerative diseases, polyunsaturated fatty acids, nutrition

## Abstract

**Objective:**

We aimed to assess the association between body mass index and polyunsaturated fatty acids to determine potential links and metabolic differences between Alzheimer's disease and dementia with Lewy bodies.

**Methods:**

We performed a cross-sectional study of enrolment data with 133 patients with mild AD (n = 75) and DLB (n = 58) from a Norwegian cohort study. We used linear regression models adjusting for age, sex, and diagnosis to explore the association between PUFA concentrations and BMI estimates.

**Results:**

In DLB patients, BMI was positively associated with downstream omega-6 PUFA, including gamma-linolenic acid (GLA; β = 0.285, *p* = 0.012), arachidonic acid (AA; β = 0.320, *p* = 0.005), and adrenic acid (β = 0.303, *p* = 0.005). BMI was also related to increased Δ6-desaturase n-6 activity (β = 0.383, *p* = 0.002) and Δ6-desaturase n-3 activity (β = 0.335, *p* = 0.011), along with reduced elongase n-6 (β = –0.396, *p* = 0.002) and elongase n-3 (β = –0.376, *p* = 0.004), suggesting increased fatty acid turnover. No significant associations were found in the AD group.

**Conclusions:**

BMI in DLB patients was positively associated with elevated downstream omega-6 metabolites and desaturase and elongase activity, compatible with higher fatty‑acid turnover and systemic inflammatory state hypothesized in DLB. These findings highlight the distinct metabolic alterations across dementia subtypes that are relevant for personalized nutritional strategies in dementia care.

## 1 Introduction

Polyunsaturated fatty acids (PUFA), including omega-3 (n-3) and omega-6 (n-6) fatty acids, are key regulators of brain function, inflammation, and systemic metabolic health [1]. Omega-3 PUFA, especially docosahexaenoic acid (DHA) and eicosapentaenoic acid (EPA), have been studied for their neuroprotective effects, with higher levels associated with reduced risk of dementia [2]. Conversely, omega-6 PUFA have been linked to pro-inflammatory pathways and a higher risk of cognitive decline [3].

Understanding lipid and metabolic interactions is increasingly relevant for geriatric nutrition, as metabolic resilience and body composition influence aging and prognosis [4]. Individuals with elevated BMI often exhibit lower omega-3 and higher levels of omega-6 PUFA, potentially driving chronic inflammation and related disorders [5].

This study investigates the association between plasma PUFA levels and baseline BMI in a Norwegian cohort of patients with AD and DLB, two of the most common causes of neurodegenerative dementia [6], to identify distinct PUFA-BMI patterns and better understand disease mechanisms and enhance personalized nutritional approaches in dementia care.

## 2 Materials & methods

### 2.1 Study design and participants

This study is a cross-sectional analysis from the Dementia Study of Western Norway (DemVest) cohort, a 12-year longitudinal study. Recruitment took place from 2005 to 2013, with participants drawn from dementia clinics and outpatient clinics in geriatric medicine and neurology [7].

Exclusion criteria included severe dementia, no dementia, delirium, bipolar or psychotic disorder, and terminal illness. Physical, neurological, psychiatric, and cognitive evaluations, as well as blood testing, were part of the baseline and annual assessments [7,8]. There was a very low attrition rate (less than 5%), and DAT-scan and CSF-amyloid were performed. All had MRI or CT scans. The diagnosis was set on an all-data available consensus meeting between geriatrics and old age psychiatrists [8].

From 196 individuals originally included in the DemVest cohort, we excluded those without available BMI data (AD: n = 36; DLB: n = 27), analysing 133 individuals (AD: n = 75; DLB: n = 58).

### 2.2 Clinical and biochemical assessments

All BMI and plasma PUFA measurements used in the present analyses were obtained at the baseline assessment (enrolment visit).

#### 2.2.1 Cognition

We evaluated global cognitive performance using the validated Norwegian version of the mini-mental state examination (MMSE) [9].

#### 2.2.2 Dementia Diagnosis

Dementia was diagnosed based on established clinical criteria. For AD, the criteria from the National Institute of Neurological and Communicative Disorders and Stroke Alzheimer's and Related Disorders Association were used [10], while the DLB diagnosis followed the 2005 consensus criteria [11]. Mild dementia was defined as a Mini-Mental State Examination (MMSE) score of ≥ 20 or a Clinical Dementia Rating (CDR) global score of 1 [9].

#### 2.2.3 Comorbidities

Comorbidities were evaluated using the Cumulative Illness Rating Scale (CIRS)[12]. The CIRS assesses health status, exploring cardiovascular-respiratory, gastrointestinal, genitourinary, musculoskeletal, and neuropsychiatric systems, with a higher score indicating higher cumulative illness [12].

#### 2.2.4 Body Mass Index (BMI)

Weight was measured without footwear, with participants wearing light indoor clothing, and recorded in kilograms (kg). Height was measured using a calibrated stadiometer and recorded in metres. Both measurements were rounded to one decimal place.

#### 2.2.5 Lipid measurements

Plasma samples were collected and stored at −80 °C until processing. PUFA plasma concentrations were quantified using standard enzymatic gas-liquid chromatography protocols as previously described [13].

The omega-6/omega-3 ratio was computed by dividing the sum of all omega-6 PUFA concentrations by the sum of all omega-3 PUFA concentrations [14].

Activity of key enzymes involved in PUFA metabolism was assessed using established proxies by estimating product-to-precursor ratios within the omega-3 (n-3) and omega-6 (n-6) pathways [15].

Specifically, Δ6 desaturase, elongase, and Δ5-desaturase indices were calculated as follows:

Omega-3 (n-3) pathway: Δ6-desaturase n-3 = 18:4n-3 / 18:3n-3; elongase n-3 = 22:5n-3 / 20:5n-3; and Δ5-desaturase n-3 = 20:5n-3 / 20:4n-3.

Omega-6 (n-6) pathway: Δ6-desaturase n-6 = 18:3n-6 / 18:2n-6; elongase n-6 = 20:3n-6 / 18:3n-6; and Δ5-desaturase n-6 = 20:4n-6 / 20:3n-6.

### 2.3 Statistical analysis

Descriptive statistics were used to calculate means, standard deviations for continuous variables, and frequencies for categorical variables. Group comparisons between the DLB and AD groups were assessed using independent Kruskal-Wallis tests for continuous variables and chi-squared tests for categorical variables. We used independent linear regression models, in which each PUFA derivative concentration was treated as the dependent variable for BMI. An interaction term with BMI and diagnosis was incorporated in the models, using the AD group as the reference. Age, sex, CIRS, MMSE, and the total PUFA concentration adjusted each model.

All continuous variables were centered around their means and scaled using standard deviations. Backward selection was applied to exclude non-relevant predictors in each model, retaining variables at a 0.10 significance level. Total PUFA was significant for α-linolenic acid, eicosapentaenoic acid, docosahexaenoic acid, total omega-3, linoleic acid, gamma-linolenic acid, arachidonic acid, adrenic acid, total omega-6, and Δ5-desaturase n-6. CIRS was retained for linoleic acid and total omega-6. Sex was retained for gamma-linolenic acid, Δ6-desaturase n-6, and elongase n-6, while age was retained for gamma-linolenic acid and Δ6-desaturase n-6. Ratio omega-6/omega-3, Δ6-desaturase n-3, elongase n-3, and Δ5-desaturase n-3 did not retain any of these additional predictors.

Primary analyses relating BMI to PUFA profiles were cross-sectional and based on baseline measurements, whereas longitudinal analyses were restricted to cognitive outcomes due to the absence of repeated PUFA assessments. Exploratory longitudinal analyses were conducted using linear mixed-effects models with MMSE and CDR–Sum of Boxes as outcomes. Models included random intercepts and slopes for time and examined baseline levels and cognitive change in relation to individual fatty acids, adjusted for education, BMI, diagnosis × time, and the fatty acid of interest. Fatty acids were log-transformed and standardised before analysis. Results are reported in the Supplementary Material.

Estimated slopes for AD and DLB were reported for each model. To reduce the risk of false findings due to multiple comparisons we applied a False discovery rate (FDR) correction to p-values using the Benjamini–Hochberg procedure [16]. The significance level was set at 0.05 for hypothesis testing. Data processing, modelling, and plots were carried out using R version 4.2.3.

## 3 Results

A total of 133 participants (75 AD and 58 DLB) were included, with characteristics summarized in Table 1. A higher proportion of females was found in the AD group (73%) compared to the DLB group 40% (*p* < 0.0002). The DLB group exhibited higher CIRS (6.90 ± 2.54) than the AD group (5.47 ± 2.50; *p* < 0.0001). There was no statistically significant difference between the two groups in age, MMSE, and mean BMI.

**Table 1:** Sample characteristics and bivariate analysis.

	AD	DLB	Total	P-value
	n (%) or mean ± sd			
Total	75 (56.4)	58 (43.6)	133 (100)	

Sex (female)	55 (73.3)	23 (39.7)	73 (58.6)	0.0002
Age	74.4 ± 7.56	75.1 ± 5.89	74.7 ± 6.87	0.9457
CIRS	5.47 ± 2.50	6.90 ± 2.54	6.03 ± 2.60	< 0.0001
MMSE	23.5 ± 2.43	23.7 ± 3.08	23.6 ± 2.72	0.4822
BMI	24.1 ± 4.12	25.0 ± 4.15	24.5 ± 4.14	0.3465

α linolenic acid	29.0 ± 14.5	25.4 ± 9.27	27.4 ± 12.5	0.2782
Eicosapentaenoic acid	58.1 ± 39.6	61.1 ± 37.7	59.4 ± 38.6	0.6797
Docosahexaenoic acid	127.7 ± 48.9	129.6 ± 45.0	124.5 ± 47.1	0.6981
Total Omega-3	214.8 ± 84.5	216.2 ± 82.4	215.4 ± 83.2	0.9602
Linoleic acid	987.7 ± 246.7	894.1 ± 204.7	946.6 ± 233.1	0.0082
Gamma-Linolenic acid	14.1 ± 7.85	10.6 ± 6.84	12.6 ± 7.60	0.0048
Arachidonic acid	213.7 ± 53.2	191.5 ± 40.0	204.0 ± 49.0	0.0088
Adrenic acid	5.70 ± 1.99	4.89 ± 2.03	5.35 ± 2.04	0.0080
Total Omega-6	1269.9 ± 247.8	1142.3 ± 231.0	1214.2 ± 263.5	0.0019
Ratio Omega-6/Omega-3	6.72 ± 2.75	5.92 ± 2.23	6.37 ± 2.56	0.0688
Δ6-desaturase n-3	0.05 ± 0.02	0.05 ± 0.03	0.05 ± 0.03	0.9205
Elongase n-3	5.86 ± 3.13	5.39 ± 2.92	5.66 ± 3.04	0.3476
Δ5-desaturase n-3	10.1 ± 6.89	12.3 ± 7.04	11.0 ± 7.02	0.0166
Δ6-desaturase n-6	0.02 ± 0.01	0.01 ± 0.01	0.01 ± 0.01	0.0341
Elongase n-6	4.68 ± 1.81	5.23 ± 2.15	4.92 ± 1.98	0.2363
Δ5-desaturase n-6	4.13 ± 1.26	4.54 ± 1.38	4.31 ± 1.32	0.0271

Descriptive characteristics and plasma fatty acid profiles of participants with Alzheimer’s disease (AD) and dementia with Lewy bodies (DLB). Values are presented as mean ± standard deviation (SD) for continuous variables and n (%) for categorical variables. P-values were derived from independent Kruskal-Wallis test for continuous variables and chi-square tests for categorical variables. PUFA abbreviations: α-linolenic acid (ALA), γ-linolenic acid (GLA), arachidonic acid (AA). Proxy enzyme activity indices (Δ5- and Δ6-desaturase, elongase) were calculated as product-to-precursor ratios within each pathway.

In bivariate analyses (Table 1), participants with DLB showed significantly lower concentrations of linoleic acid (LA) *p* = 0.0082, gamma-linolenic acid (GLA) *p* = 0.0048, arachidonic acid (AA) *p* = 0.0088, adrenic acid *p* = 0.008, total omega-6 PUFA *p* = 0.0019, indices for Δ5-desaturase (n-3) *p* = 0.0166, Δ6-desaturase (n-6) *p* = 0.0341, and Δ5-desaturase (n-6) *p* = 0.0271 compared with those with AD *(p < 0.05*) (Table 1). In the bivariate analysis, no significant differences in total or individual omega-3 PUFA, the omega-6/omega-3 ratio were observed between the AD and DLB groups (Table 1).

### 3.1 PUFA-BMI associations in AD and DLB

**Fig. 1:**
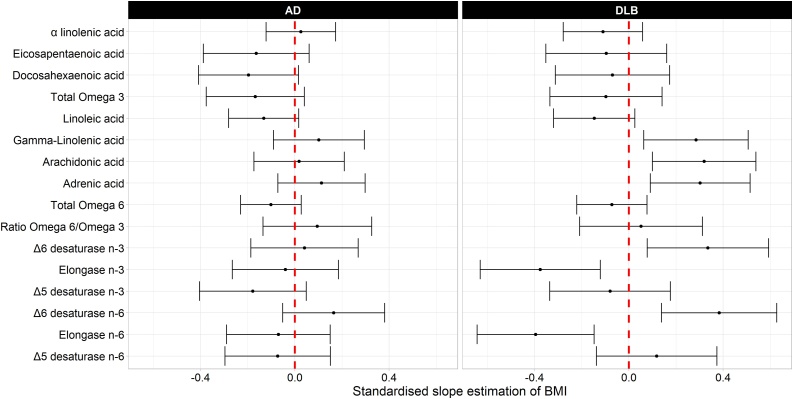
Associations Between Body Mass Index and Plasma PUFA Profiles in Alzheimer’s Disease and Dementia with Lewy Bodies. β-coefficients (dots) and 95 % confidence intervals (horizontal bars) from multiple linear regression models. Each coefficient was estimated at the mean level of the adjusted variables. Models are adjusted for age, sex, MMSE, CIRS, and total PUFA. The red dashed line indicates the null (β = 0). Positive β indicates higher metabolite levels with increasing BMI.

In the DLB group, BMI was positively associated with several downstream omega-6 PUFA, including gamma-linolenic acid (GLA; β = 0.285, *p* = 0.012), arachidonic acid (AA; β = 0.320, *p* = 0.005) and adrenic acid (β = 0.303, *p* = 0.005) (Table 2, Fig. 1). We also found significant associations for enzymatic activity indices including Δ6-desaturase n-6 (β = 0.383, *p* = 0.002) and elongase n-6 (β = –0.396, *p* = 0.002). BMI was also associated with higher Δ6-desaturase n-3 activity (β = 0.335, *p* = 0.011) and lower elongase n-3 activity (β = –0.376, *p* = 0.004) (Table 2).

**Table 2:** Estimated Associations Between Body Mass Index and Plasma PUFA Levels and Enzyme Activity Indices.

	Est.	Std. Err.	P-value	Adj. P		Est.	Std. Err.	P-value	Adj. P
	AD					LBD			
α linolenic acid	0.030	0.070	0.736	0.760		0.110	0.090	0.198	0.396
Eicosapentaenoic acid	0.160	0.110	0.180	0.384		0.100	0.130	0.462	0.643
Docosahexaenoic acid	0.197	0.107	0.068	0.272		0.070	0.123	0.572	0.678
Total Omega 3	0.168	0.105	0.113	0.298		0.097	0.120	0.421	0.612
Linoleic acid	0.133	0.075	0.080	0.284		0.147	0.087	0.094	0.298
Gamma-Linolenic acid	0.102	0.098	0.299	0.532		0.285	0.112	**0.012**	0.055
Arachidonic acid	0.018	0.097	0.855	0.855		0.320	0.111	**0.005**	**0.032**
Adrenic acid	0.113	0.094	0.231	0.435		0.303	0.107	**0.005**	**0.032**
Total Omega 6	0.102	0.065	0.119	0.298		0.072	0.075	0.340	0.573
Ratio Omega 6/Omega 3	0.095	0.117	0.416	0.612		0.052	0.132	0.695	0.760
Δ6 desaturase n-3	0.041	0.115	0.726	0.760		0.335	0.130	**0.011**	0.055
Elongase n-3	0.040	0.114	0.724	0.760		0.376	0.013	**0.004**	**0.032**
Δ5 desaturase n-3	0.179	0.114	0.121	0.298		0.080	0.129	0.539	0.663
Δ6 desaturase n-6	0.165	0.109	0.135	0.309		0.383	0.124	**0.002**	**0.032**
Elongase n-6	0.070	0.112	0.530	0.663		0.396	0.125	**0.002**	**0.032**
Δ5 desaturase n-6	0.073	0.113	0.517	0.663		0.118	0.129	0.362	0.579

Standardized β-coefficients (Est.), standard errors (Std. Err), raw p-values and corrected using the false discovery rate (FDR) method (Adj. P). from multiple linear regression models examining associations between body mass index (BMI) and plasma polyunsaturated fatty acids (PUFA) in participants with Alzheimer’s disease (AD) and dementia with Lewy bodies (DLB). Each model was adjusted for age, sex, Mini-Mental State Examination (MMSE) score, Cumulative Illness Rating Scale (CIRS), and total PUFA concentration, with diagnosis and its interaction with BMI included to estimate group-specific slopes. Δ5- and Δ6-desaturase and elongase indices were calculated as product-to-precursor ratios within the n-3 and n-6 pathways.

In contrast, no significant associations were found between BMI and PUFA concentrations in the AD group. Neither total omega-3 nor total omega-6 concentrations, nor the omega-6/omega-3 ratio, showed significant relationships with BMI in AD or DLB after adjustment for covariates (Table 2). After false discovery rate (FDR) correction for multiple testing, the associations with gamma-linolenic acid and Δ6-desaturase n-3 did not remain statistically significant (Table 2).

### 3.2 Exploratory longitudinal analyses

In exploratory longitudinal mixed-effects models, no statistically significant associations were observed between individual plasma fatty acids and cognitive trajectories as measured by MMSE or CDR–Sum of Boxes. This was consistent across omega-3 and omega-6 fatty acids, fatty acid ratios, and enzymatic activity indices, both at baseline and in relation to cognitive change over time. Detailed results are presented in Supplementary Table S1.

## 4 Discussion

Our findings show correlations between higher BMI in the DLB group and elevated levels of downstream omega-6 PUFA, including GLA, AA, and adrenic acid (Table 2, Fig. 1). Increased intake of omega-6 PUFA has been related to dementia risk [17]. Neither total omega-3, omega-6 concentrations nor the overall omega-6/omega-3 ratio were significantly associated with BMI (Table 2). The lack of association at the total PUFA level may reflect heterogeneity within PUFA families.

Although DLB patients show a pronounced BMI decline [18], within this group, higher BMI was associated with pro-inflammatory omega-6 metabolites, which may be consistent with systemic metabolic strain and a more aggressive clinical phenotype compared to AD [19]. These patterns align with evidence of systemic inflammatory states and altered lipid metabolism, potentially linked to α-synuclein pathology [20].

Evidence from studies with other α-synucleinopathies, like Parkinson’s disease, shows similar lipid-related mechanisms, with dysregulated AA metabolism [21], and excess omega-6 exposure linked to higher incidence risk [22]. Experimental and postmortem studies show that altered PUFA composition can promote lipid peroxidation, reactive oxygen species production (ROS), and α-synuclein oligomerization, indicating disrupted PUFA profiles in Lewy body-related neurodegeneration [20,23,24]. In contrast, no significant associations were observed between BMI and PUFA concentrations in the AD group. This may indicate a weaker link between body composition and peripheral fat metabolism in this dementia subtype. This lack of association highlights a potential difference in metabolic regulation between DLB and AD, emphasizing that PUFA can significantly alter α-synuclein’s molecular structure and aggregation [25,26].

DLB is also characterized by a systemic deterioration, including malnutrition and frailty [27]. Low omega-3 levels have been linked to increased frailty [28], highlighting the relevance of metabolic vulnerability in this group. Monitoring body composition rather than relying solely on BMI may better capture the interplay between metabolic disturbances and systemic degeneration in DLB [29].

These findings suggest an interaction between PUFA metabolism and body composition in dementia, particularly in DLB, reflecting metabolic dysregulation. While these mechanistic hypotheses are promising, this study has several limitations. The lack of a healthy control group and potential selection bias may affect generalizability. Additionally, dietary intake information was not provided, and our cross-sectional study design could have masked dynamic PUFA-BMI relationships detectable in longitudinal analyses. Consistent with this, exploratory longitudinal analyses did not identify significant associations between plasma fatty acids and cognitive trajectories, and longitudinal PUFA measurements were unavailable, limiting our ability to assess within-person metabolic change over time. Moreover, sample size and variability could have mediated the lack of significant associations in the AD group. Future longitudinal studies should examine whether these lipid signatures reflect disease progression or response to nutritional and supplementation interventions.

## 5 Conclusions

Our study identifies that higher BMI in patients with DLB is associated with elevated downstream omega-6 metabolites and altered desaturase and elongase activity, suggesting increased fatty acid turnover and systemic metabolic stress. In contrast, no significant associations were observed in the AD group. These findings indicate that lipid and body composition relationships across dementia are subtype-specific and may reflect divergent metabolic or inflammatory mechanisms in DLB, which may be relevant when considering personalized nutritional strategies in aging and dementia care.

## CRediT authorship contribution statement

Conceptualization: N.C.P., M.G.B.

Methodology: N.C.P., D.A., A.O.V.-M.

Formal analysis: D.A.T.R.

Investigation: N.C.P., J.R.C., A.J.J.

Data curation: D.A.T.R., N.C.P.

Writing original draft: N.C.P.

Writing review and editing: N.C.P., M.G.B., J.R.C., A.J.J., G.E.B., R.K.B., D.A., A.O.V.-M.

Visualization: N.C.P., D.A.T.R.

Supervision: D.A., A.O.V.-M., G.E.B.

Funding acquisition: D.A.

## Funding statement

DemVest study was funded by the Norwegian government through hospital owner Helse Vest (Western Norway Regional Health Authority), grant number 911973. It was also supported by the NIHR Biomedical Research Centre at South London and Maudsley NHS Foundation Trust, as well as King's College London (UK).

## Ethical statements

Participants provided informed consent before inclusion in the study. The regional ethics committee approved the DemVest study (approval code 2010/633) for the collection of medical data. All information was handled and kept following national health and data privacy protocols.

## Declaration of Competing Interest

D.A. has received research support and/or honoraria from Astra‐Zeneca, H. Lundbeck, Novartis Pharmaceuticals, Evonik, and GE Health and has served as paid consultant for H. Lundbeck, Axovant, Eisai, Heptares, Mentis Cura, Eli Lilly, and Biogen. A-O.V-M. Paid consultant for Eisai.

## Data availability statement

The participants of this study did not give written consent for their data to be shared publicly; therefore, due to the sensitive nature of the research, supporting data are not available.

## Declaration of Generative AI and AI-assisted technologies in the writing process

Microsoft Copilot, which uses OpenAI’s GPT-4 model, was used for language improvement and text clarity only. No AI tools were used for study design, data analysis, statistical modeling or interpretation of the results.
