# Corrigendum to “Distinct associations between body mass index and polyunsaturated fatty acids in dementia with Lewy Bodies and Alzheimer’s disease” [J Nutr Health Aging, 30 (2026) 100810]

**DOI:** 10.1016/j.jnha.2026.100974

**Published:** 2026-09-06

**Authors:** Nicolás Castellanos-Perilla, Miguel Germán Borda, Joyce Ruifen Chong, Diego Alejandro Tovar-Rios, Alberto Jaramillo-Jimenez, Rolf K. Berge, George E. Barreto, Dag Aarsland, Audun Osland Vik-Mo

**Affiliations:** a Department of Clinical Medicine, University of Bergen, Bergen, Norway; b Centre for Age-Related Medicine (SESAM), Stavanger University Hospital, Stavanger, Norway; c Department of Neurobiology, Care Sciences and Society, Karolinska Institutet, Stockholm, Sweden; d Department of Neurology, Clínica Universidad de Navarra, Pamplona, Spain; e Department of Pharmacology, Yong Loo Lin School of Medicine, National University of Singapore, Singapore, Singapore; f Memory, Aging and Cognition Centre, National University Health Systems, Singapore, Singapore; g Centre for Healthy Brain Ageing, Institute of Psychiatry, Psychology, and Neuroscience, King's College London, London, United Kingdom; h L-BioStat, KU Leuven, Leuven, Belgium; i Grupo de Investigación en Estadística Aplicada - INFERIR, Facultad de Ingeniería, Universidad del Valle, Santiago de Cali, Valle del Cauca, Colombia; j Prevención y Control de la Enfermedad Crónica - PRECEC, Facultad de Salud, Universidad del Valle, Santiago de Cali, Valle del Cauca, Colombia; k Grupo de Neurociencias de Antioquia (GNA), Facultad de Medicina, Universidad de Antioquia, Medellín, Colombia; l Department of Clinical Science, University of Bergen, Bergen, Norway; m Department of Biological Sciences, University of Limerick, Limerick, Ireland

The authors regret the following error in the original publication: the name of the last author was incorrectly listed as “Audun Osland Vik-M” instead of the correct name, “Audun Osland Vik-Mo”.

The authors would like to apologise for any inconvenience caused.

