# Supplementary material for: Distinct associations between body mass index and polyunsaturated fatty acids in dementia with Lewy Bodies and Alzheimer’s disease
Source: J Nutr Health Aging. 2026 Feb 21;30(4):100810. doi: 10.1016/j.jnha.2026.100810 (PMC12945632; doi:10.1016/j.jnha.2026.100810)
Supplement: Supplementary file 1 [file mmc1.docx]

**Supplementary Table 1. Exploratory longitudinal associations between plasma fatty acids and cognitive outcomes (MMSE and CDR-Sum of Boxes)**

| Análisis longitudinal de MMSE y CDR |  | |  |  | |  | |  |  |
| --- | --- | --- | --- | --- | --- | --- | --- | --- | --- |
|  | **Est.** | **Std. Err.** | **P-value** | **Adj. P** |  | **Est.** | **Std. Err.** | **P-value** | **Adj. P** |
|  | *MMSE* | | | |  | *CDR* | | | |
| α linolenic acid | 0,021 | 0,058 | 0,712 | 0,986 | | 0,002 | 0,050 | 0,970 | 0,970 |
| Eicosapentaenoic acid | 0,060 | 0,057 | 0,300 | 0,986 | | -0,028 | 0,050 | 0,583 | 0,848 |
| Docosahexaenoic acid | 0,043 | 0,058 | 0,457 | 0,986 | | -0,047 | 0,051 | 0,357 | 0,710 |
| Total Omega 3 | 0,052 | 0,058 | 0,372 | 0,986 | | -0,040 | 0,050 | 0,430 | 0,710 |
| Linoleic acid | 0,001 | 0,058 | 0,979 | 0,986 | | 0,042 | 0,050 | 0,412 | 0,710 |
| Gamma-Linolenic acid | -0,024 | 0,057 | 0,672 | 0,986 | | 0,008 | 0,051 | 0,879 | 0,944 |
| Arachidonic acid | -0,010 | 0,057 | 0,864 | 0,986 | | -0,007 | 0,049 | 0,885 | 0,944 |
| Adrenic acid | -0,043 | 0,059 | 0,469 | 0,986 | | 0,040 | 0,052 | 0,444 | 0,710 |
| Total Omega 6 | -0,038 | 0,058 | 0,948 | 0,986 | | 0,043 | 0,051 | 0,397 | 0,710 |
| Ratio Omega 6/Omega 3 | -0,055 | 0,059 | 0,358 | 0,986 | | 0,064 | 0,051 | 0,216 | 0,710 |
| Δ6 desaturase n-3 | -0,007 | 0,054 | 0,903 | 0,986 | | -0,014 | 0,048 | 0,771 | 0,944 |
| Elongase n-3 | 0,032 | 0,052 | 0,575 | 0,986 | | 0,077 | 0,049 | 0,119 | 0,661 |
| Δ5 desaturase n-3 | 0,027 | 0,059 | 0,649 | 0,986 | | -0,080 | 0,051 | 0,124 | 0,661 |
| Δ6 desaturase n-6 | -0,024 | 0,056 | 0,669 | 0,986 | | -0,010 | 0,050 | 0,838 | 0,944 |
| Elongase n-6 | 0,028 | 0,056 | 0,612 | 0,986 | | 0,044 | 0,049 | 0,369 | 0,710 |
| Δ5 desaturase n-6 | 0,001 | 0,056 | 0,986 | 0,986 | | -0,077 | 0,050 | 0,124 | 0,661 |

β coefficients from exploratory linear mixed-effects models. Fatty acids were log-transformed and standardised (mean = 0, SD = 1); thus, β values reflect the change in MMSE or CDR Sum of Boxes associated with a 1-SD increase in the fatty acid level. Models included random intercepts and slopes for time and were adjusted for education, BMI, diagnosis × time, and the individual fatty acid.
